# COMPARATIVE STUDY ON LIVER TRANSPLANTATION WITH AND WITHOUT HEPATOCELLULAR CARCINOMA WITH CIRRHOSIS: ANALYSIS OF MELD, WAITING TIME AND SURVIVAL

**DOI:** 10.1590/0102-6720201600010006

**Published:** 2016

**Authors:** Alexandre Coutinho Teixeira de FREITAS, Rafael Shinmi SHIGUIHARA, Ruan Teles MONTEIRO, Thiago Linck PAZETO, Júlio Cezar Uili COELHO

**Affiliations:** Hospital de Clínicas, Liver Transplantation Unit, Federal Universtiy of Paraná, Curitiba, Paraná, Brazil

**Keywords:** Carcinoma, hepatocellular, Liver transplantation, Liver cirrhosis, Survival analysis

## Abstract

***Background*:**

Liver transplantation is the usual treatment for hepatocellular carcinoma.

**Aim:**

To analyze the MELD score, waiting time and three month and one year survival for liver transplantation in cirrhotic patients affected by hepatocellular carcinoma or not.

***Methods*:**

This was a retrospective, observational and analytical study of 93 patients submitted to liver transplantation.

***Results*:**

There were 28 hepatocellular carcinoma and 65 non-hepatocellular carcinoma patients with no differences related to age and sex distribution. The main causes of cirrhosis on hepatocellular carcinoma were hepatitis C virus (57.1%) and hepatitis B virus (28.5%), more frequent than non-hepatocellular carcinoma patients, which presented 27.7% and 4.6% respectively. The physiological and exception MELD score on hepatocellular carcinoma were 11.9 and 22.3 points. On non-hepatocellular carcinoma, it was 19.4 points, higher than the physiological MELD and lower than the exception MELD on hepatocellular carcinoma. The waiting time for transplantation was 96.2 days for neoplasia, shorter than the waiting time for non-neoplasia patients, which was 165.6 days. Three month and one year survival were 85.7% and 78.6% for neoplasia patients, similar to non-neoplasia, which were 77% and 75.4%.

**Conclusion:**

Hepatocellular carcinoma patients presented lower physiological MELD score, higher exception MELD score and shorter waiting time for transplantation when compared to non-hepatocellular carcinoma patients. Three month and one year survival were the same between the groups.

## Abstract (pt)

**Racional:**

O tratamento habitual do carcinoma hepatocelular é o transplante hepático.

**Objetivo:**

Analisar o MELD, o tempo em lista de espera e a sobrevida em três meses e em um ano de pacientes submetidos ao transplante hepático cadavérico e divididos em dois grupos: portadores e não portadores de carcinoma hepatocelular.

**Método:**

Estudo analítico, observacional e retrospectivo de 93 pacientes transplantados.

**Resultados:**

Os grupos foram similares em relação ao sexo e à idade. Os 28 pacientes com a neoplasia apresentaram maior frequência de vírus da hepatite C (57,1%) e da hepatite B (28,5%) em relação aos 65 não portadores, que foi de 27,7% e 4,6% respectivamente. O MELD fisiológico e corrigido dos portadores de carcinoma hepatocelular foi de 11,9 e 22,3 pontos, enquanto que o dos pacientes sem foi de 19,4 pontos, maior do que o MELD fisiológico e menor do que o MELD corrigido dos portadores. O tempo em lista de espera dos com a neoplasia foi de 96,2 dias, menor do que a dos sem a ela que foi de 165,6 dias. A sobrevida em três meses e em um ano dos pacientes acometidos pela neoplasia foi de 85,7% e 78,6%, igual à sobrevida dos não acometidos que foi de 77% e 75,4%.

**Conclusão:**

Os portadores de carcinoma hepatocelular apresentaram menor MELD fisiológico, menor tempo em lista e maior MELD corrigido em relação aos não portadores. A sobrevida em três meses e em um ano foi igual entre os grupos.

## INTRODUCTION

Hepatocellular carcinoma (HCC) is the third leading cause of death from neoplasia worldwide, affecting one million people, with about five hundred thousand deaths annually^1^. In Brazil, it was responsible for about 52,560 deaths from 2001 to 2010, being the second leading cause for liver disease. Its incidence is increasing annually in Western countries due to the higher number of patients infected with hepatitis C and B, which are present in approximately 90% of cases^1^^,^ 9 ^,^ 20.

Approximately 80% of HCC patients have concomitant cirrhosis^16^^,^ 21. Those with liver cirrhosis have a 20% chance of developing the tumor in five years^6^ **.** This high association defines the population of cirrhotic patients as risky, which must be followed through periodic screening, allowing the early diagnosis of the tumor^24^.

Surgical resection is not a viable option for a large numbers of cirrhotic patients with HCC. The functional hepatic reserve may not be enough to tolerate and compensate the removal of the hepatic parenchyma^30^^,^ 33. Moreover, factors such as portal hypertension make the surgical risk very high. Given these facts, the transplant is considered the therapy of choice for non-candidates for resection, because besides the removal of the tumor, it restores liver function and reduces the risk of relapse.

The results are excellent for hepatic transplantation in patients with solitary nodules up to 5 cm in diameter or up to three nodules smaller than 3 cm (Milan criteria). The five-year survival after transplantation in this situation is up to 73%^3^^,^ 18 ^,^ 19 ^,^ 22 ^,^ 23 ^,^ 26. 

In 2006 it occurred in Brazil the implementation of the MELD (Model for End-Stage Liver Disease), which determines the waiting time for liver transplantation based on the severity of cirrhosis. Situations such as the diagnosis of HCC are also prioritized in this model^9^. The liver transplantation candidates with HCC within the Milan criteria are given special score (exception MELD) due to the projected risk of neoplasia progression of neoplasia^10^.

The aim of this study was to analyze the MELD score, waiting time and survival in three months and one year in cirrhotic patients with and those without HCC undergoing cadaveric liver transplantation.

## METHODS

This study was approved by Ethics Committee for Human Research of the Clinical Hospital of the Federal University of Parana, Curitiba, Brazil.

From June 2006 to October 2014 medical records of 138 patients submitted to cadaveric liver transplantation were reviewed.

Inclusion criteria were patients with cirrhosis only or cirrhosis with HCC within the Milan criteria at the time of transplant. Exclusion criteria were pediatric patients (children under 12 years-old); those undergoing simultaneous liver and kidney transplantation, living related liver transplantation; individuals with HCC without cirrhosis; individuals with incomplete data records; and patients included on the waiting list for a liver before the implementation of the MELD score. The information obtained from the receptors were: waiting time for a liver, MELD score at transplantation (physiological MELD), exception MELD under special circumstances (patients with HCC) and patient cumulative survival after three months and after one year the transplantation. There were also collected secondary information such as gender, age and cause of cirrhosis.

Cirrhotic patients underwent follow-up protocol with ultrasound and alpha-fetal protein dosage semiannually. In suspected cases of HCC, the diagnosis was confirmed by magnetic resonance imaging, computed tomography, or liver biopsy. Patients were divided into two groups: cirrhotic patients with HCC and cirrhotic patients without HCC.

All individuals with HCC submitted to cadaveric liver transplantation were in accordance with the Milan criteria at the time of inclusion in the transplantation list and at the time of the procedure. For individuals who were outside the criteria it was performed cytoreduction therapy (downstaging) through procedures such as chemoembolization and alcoholysis in order to make it possible their inclusion in the waiting list. When necessary, bridge therapy was used through the same methods for cytoreduction in order to keep the tumor within the criteria. Patients who suffered progression of the tumor size or number beyond Milan criteria, as well as those who died during waiting time for a liver were excluded from the study. MELD score was calculated based on serum bilirubin, serum creatinine and INR 8. 

According to Brazilian legislation, exceptional MELD score can be considered for patients with HCC. After the diagnosis within the Milan criteria the nodules must be analyzed based on radiological, laboratorial or pathological findings. Radiological findings for exceptional MELD score were: a) two overlapping images among three techniques (ultrasonography with microbubbles, computed tomography, nuclear magnetic resonance) demonstrating nodule with a diameter equal to or greater than 2 centimeters and with hypervascularization; b) a three-phase imaging method (ultrasonography with microbubbles, computed tomography, nuclear magnetic resonance) demonstrating nodule with a diameter equal to or greater than 2 centimeters, showing hypervascularization during the arterial phase and washout of the contrast during the portal phase. Laboratorial findings for exceptional MELD score were considered together with one method of the aforementioned demonstrating nodule with a diameter equal to or greater than 2 centimeters with hypervascularization. In this situation, exceptional MELD score was considered if serum alpha-fetoprotein level was greater than 200 ng/ml. The anatomopathological criteria was used for nodules equal to or greater than 1 centimeter and less than 2 centimeters in diameter. In this situation the diagnosis was confirmed by guided biopsy. Nodules with a diameter smaller than 1 centimeter were followed up with imaging techniques and did not receive exceptional MELD score. 

Exceptional MELD score was initially 20 points. After 3 months waiting for a liver the score was automatically increased to 24 and after 6 months it was increased to 29.

Student t test was used for continuous variables and Chi-square test for proportional variables. Mann-Whitney test was used for MELD score analysis. Kaplan-Meier curves and Cox-Mantel test were used for survival analysis. A 5% significance level was considered (p ≤ 0.05).

## RESULTS

One hundred and thirty eight patients were analyzed, 45 were excluded because they were not within the selection criteria. Ninety-three were included: 28 HCC patients and 65 non-HCC patients. The average age at transplantation was 51.1 years (27-69 years), 73 patients were male (78.5%) and 20 females (21.5%).There were no differences regarding gender and age in both groups (Table 1).

**TABLE 1:** - General characteristics of HCC and non-HCC patients submitted to transplantation

**Characteristics**	**HCC Patients (n=28)**	**Non-HCC Patients (n=65)**	**P Value**
Transplantation Age (years)	53,7±9,1	50±11,3	>0,05
Gender (male / female)	21,4%/78,5%	21,5%/78,4%	>0,05
Etiology			
HCV	12 (42,9%)	14 (21,53%)	0,035
HBV	6 (21,42%)	3 (4,6%)	0,011
Cryptogenic	3	2	-
HCV + HBV	2	0	-
HCV + alcohol	2	4	-
PBC	1	1	-
Adenomatosis	1	0	-
AIH + CBP	1	0	-
Others*	0	41	-

HCC = hepatocellular carcinoma; HCV = Hepatitis C virus; HBV = hepatitis B virus; PBC = primary biliary cirrhosis; AIH = autoimmune hepatitis. *Alcoholic cirrhosis; non-alcoholic hepatic steatosis ; secondary biliary cirrhosis; alpha 1-antitrypsin deficiency; choledochal cyst; Hemochromatosis + non-alcoholic hepatic steatosis; drug-induced hepatitis; fulminant hepatitis;

The causes of cirrhosis were: hepatitis C virus (28%), alcoholic hepatitis (17.2%), hepatitis B virus (9.7%), non-alcoholic steatohepatitis (7,5%), hepatitis C virus associated with alcoholic hepatitis (6.5%), cryptogenic cirrhosis (5.4%) and others (25.7%). Patients with HCC had higher frequency of hepatitis C (57.1%) and hepatitis B (28.5%) compared to non-HCC patients, which was 27.7% and 4.6% respectively (Table 1). 

Pre-transplantation MELD score was 17.1 ± 5.7 points. Patients with HCC had lower pre-transplantation MELD score (11.8 ± 3.5) compared to non-HCC patients (19.4 ± 5.0; p <0.05, Table 2). Exception MELD score in patients with neoplasia was 22.3 ± 3.3, higher than the physiological MELD (19.4 ± 5.0) from non-HCC patients (p <0.05). 

**TABLE 2:** - Comparative analysis of MELD score, exception MELD score and time waiting for a liver on HCC and non-HCC patients

**Characteristics**	**HCC Patients (n=28)**	**Non-HCC Patients (n=65)**	**P Value**
Pre-Transplantation MELD score	11,8±3,5	19,4±5,0	<0,05
Exception MELD score	22,3±3,3	-	<0,05
Time waiting for a liver (days)	96,2±93,5	165±231,5	0,02

HCC - hepatocellular carcinoma.

General time waiting for a liver was 144.7 ± 202.1 days. For HCC patients it was 96.2 ± 93.5 days, lower than the observed for non-HCC patients, which was 165 ± 231.5 days (p = 0.02, table 2).

**FIGURE 1:**
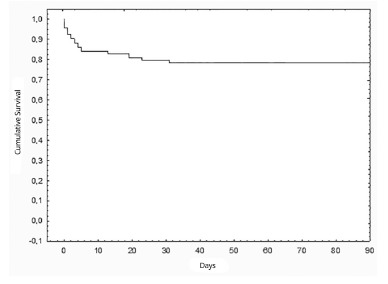
- Overall three months cumulative survival after liver transplantation

**FIGURE 2:**
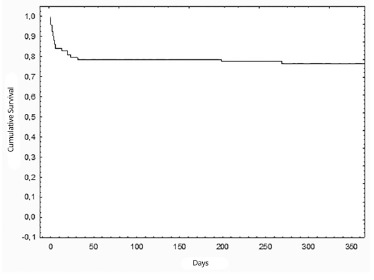
- Overall one year cumulative survival after liver transplantation

Overall three months cumulative survival was 79.6% (Figure 1) and one year cumulative survival was 76.3% (Figure 2).

**FIGURE 3:**
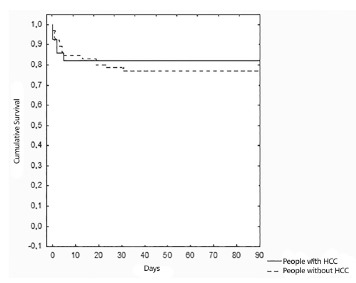
- Three months cumulative survival for patients undergoing liver transplantation according to the presence or absence of HCC

**FIGURE 4:**
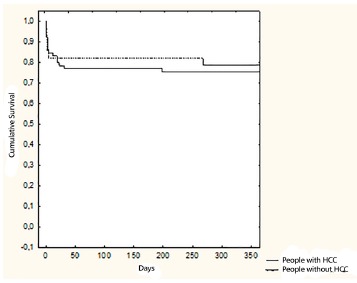
- One year cumulative survival for patients undergoing liver transplantation according to the presence or absence of HCC

Kaplan-Meier curves on Figures 3 and 4 show three months and one year cumulative survival for patients with and without HCC individually. According to Figure 3, three months survival for patients with HCC was 82.1%, similar to that for non-HCC patients which was 76.9% (p = 0.6). In Figure 4, one-year survival for patients with HCC was 78.5%, also similar to that for non-HCC patients, which was 75.3% (p = 0.7). 

## DISCUSSION

Initially, in Brazil, patients were placed on the liver transplantation waitlist in chronological (first come, first served) order. Since 2006, MELD has being used as criteria for the distribution of liver grafts. In the United States, this was introduced in 2002. This system assesses the severity of cirrhosis and predicts mortality in 90 days, allowing liver transplantation for the most severe cases. From the beginning, it was observed that this system is flawed in determining mortality in some situations that do not exhibit significant impairment of liver function, including HCC. HCC diagnosis is usually performed in cirrhotic livers subjected to a screening program with imaging and laboratorial exams. This must begin very early over the development of the disease where there has been no significant alteration in liver function. Recently, studies demonstrated that the physiological MELD score of HCC patients is lower than those without HCC^23^. The same was observed in the present study. 

Although these patients have lower physiological MELD score, they have high mortality regardless of liver function, related to tumor progression. Also, they are more prone to removal from waitlist because of clinical decompensation or development of metastases^10^^,^ 32 **.** It is believed that this risk ranges from 20% to 50%^7^^,^ 14.

Due to this fact, patients with HCC are framed in an exceptional situation in the organ allocation system that allows a greater chance to access liver transplantation. This resulted in an increased number of transplantations in patients with HCC^4^^,^ 9. This exceptional situation determines extra score to patients with HCC, allowing them to compete for an organ under more favorable conditions. This study showed that this extra score turned MELD values higher than those observed on patients without HCC.

Before the introduction of MELD score in organ allocation system, patients with HCC waited 10 to 12 months for a liver^28^. After MELD was introduced, there was a significant reduction of this time. The present study demonstrated that waiting time was significantly shorter for patients with HCC than for non-HCC patients. Similar results were found by other authors^5^. One study showed 55.6% reduction in the number of days on the waiting list^23^. Given this fact, the death rate while waiting for a liver significantly decreased^23^^,^ 29. 

After several years of its implementation in Brazil, a critical analysis should be made on this extra scoring system for cases of HCC. This has already been done in the United States and has shown that instead of making fair the competition for an organ, patients classified as exceptions, among them those with HCC, have presented numerous advantages. One study showed that such patients have greater access to a liver for transplantation^19^. The procedure was performed in 79% of patients with some exception, among them HCC, and in 40% of patients without exception. In these, time on the waitlist was 180% higher. The mortality rate while on the waiting list was 4% for the exception patients and 24% for those without exception. 

It is difficult to establish limits or determine an ideal situation for transplantation in these special situations. According to the current rule in Brazil, all transplant patients in this study met the Milan criteria for transplantation indication. The classic study of Mazzafero demonstrated that five-year survival was much lower for patients transplanted with expanded Milan criteria 17. However, several other authors have shown excellent results, from the oncologic point of view, on transplantations done with expanded criteria. There is no doubt that liver transplantation is the best treatment alternative for selected patients who are outside the Milan criteria. The problem lies in the fact that organ donation is a limited resource in relation to its offer and the inclusion of more patients in exception situation certainly greatly reduce the likelihood to receive a graft of those who do not have it. This is a policy decision that should be periodically reviewed according to the evolution of the national transplantation program. 

The first historical results of liver transplantation were disappointing in relation to survival. The procedure was performed in patients with very advanced disease and three-year survival was 25-31%^17^^,^ 28. This fact dramatically changed over the course of time due to technical advances, immunosuppression and more refined selection of recipients. Currently, liver transplantation in patients with HCC has a good life expectancy^23^. Several authors have shown similar survival among HCC and non-HCC patients 5 ^,^ 11 ^,^ 17.

The etiology of cirrhosis is a key factor determining survival for patients undergoing liver transplantation 12 ^,^ 13. Since the 70's, it is known the association between primary liver cancer and viral hepatitis B^2^. However, hepatitis C virus is currently recognized as the main cause of HCC in the world. Some authors showed prevalence of 90% of these infections in patients with HCC.^1^The results of this study confirm the prevalence of hepatitis C and B in patients with HCC. On cirrhotic patients who did not have neoplasia, the leading etiologies were: alcoholism, hepatitis C and nonalcoholic hepatic steatosis. These findings were confirmed by other authors^27^.

Some studies determined that transplant patients infected with hepatitis C had higher mortality compared to those with cirrhosis from other causes 25 ^,^ 31. In the present study, the prevalence of cirrhosis caused by hepatitis C was higher in patients with HCC, but survival was similar. Other factors must be analyzed in this context, especially the lowest physiological MELD score in patients with HCC. This is a factor that generates expectation of better postoperative prognosis and has the potential to nullify the effects of cirrhosis etiology on survival. 

## CONCLUSION

Patients with HCC showed lower physiological MELD score and higher exception MELD score compared to non-HCC patients. Neoplasia patients had lower time waiting for a liver. There were no differences on three months and one year survival among HCC patients and non-HCC patients.
